# Knockout of receptor for advanced glycation end‐products attenuates age‐related renal lesions

**DOI:** 10.1111/acel.12850

**Published:** 2019-02-22

**Authors:** Thibault Teissier, Valentine Quersin, Viviane Gnemmi, Maité Daroux, Mike Howsam, Florian Delguste, Cécile Lemoine, Chantal Fradin, Ann‐Marie Schmidt, Christelle Cauffiez, Thierry Brousseau, François Glowacki, Frédéric J. Tessier, Eric Boulanger, Marie Frimat

**Affiliations:** ^1^ U995 ‐ Lille Inflammation Research International Center INSERM, CHU Lille, University of Lille Lille France; ^2^ Department of Nephrology CHU Lille Lille France; ^3^ Department of Pathology U1172 ‐ Jean‐Pierre Aubert Research Center, INSERM, CHU Lille, University of Lille Lille France; ^4^ Department of Medicine, Diabetes Research Center NYU Langone Medical Center New York New York; ^5^ EA4483 IMPECS‐IMPact of Environmental ChemicalS on Human Health CHU Lille, University of Lille Lille France; ^6^ UF8832 ‐ Biochimie Automatisée Pôle de Biologie Pathologie Génétique CHU Lille Lille France; ^7^ Department of Geriatrics CHU Lille Lille France

**Keywords:** advanced glycation end‐products, amyloidosis, chronic kidney disease, nephrosclerosis, receptor for AGEs, renal aging

## Abstract

Pro‐aging effects of endogenous advanced glycation end‐products (AGEs) have been reported, and there is increasing interest in the pro‐inflammatory and ‐fibrotic effects of their binding to RAGE (the main AGE receptor). The role of dietary AGEs in aging remains ill‐defined, but the predominantly renal accumulation of dietary carboxymethyllysine (CML) suggests the kidneys may be particularly affected. We studied the impact of RAGE invalidation and a CML‐enriched diet on renal aging. Two‐month‐old male, wild‐type (WT) and RAGE^−/−^ C57Bl/6 mice were fed a control or a CML‐enriched diet (200 μg CML/g_food_) for 18 months. Compared to controls, we observed higher CML levels in the kidneys of both CML WT and CML RAGE^−/−^ mice, with a predominantly tubular localization. The CML‐rich diet had no significant impact on the studied renal parameters, whereby only a trend to worsening glomerular sclerosis was detected. Irrespective of diet, RAGE^−/−^ mice were significantly protected against nephrosclerosis lesions (hyalinosis, tubular atrophy, fibrosis and glomerular sclerosis) and renal senile apolipoprotein A‐II (ApoA‐II) amyloidosis (*p* < 0.001). A positive linear correlation between sclerosis score and ApoA‐II amyloidosis score (*r* = 0.92) was observed. Compared with old WT mice, old RAGE^−/−^ mice exhibited lower expression of inflammation markers and activation of AKT, and greater expression of *Sod2* and SIRT1. Overall, nephrosclerosis lesions and senile amyloidosis were significantly reduced in RAGE^−/−^ mice, indicating a protective effect of RAGE deletion with respect to renal aging. This could be due to reduced inflammation and oxidative stress in RAGE^−/−^ mice, suggesting RAGE is an important receptor in so‐called inflamm‐aging.

## 1 INTRODUCTION

Kidney function declines with age (Bolignano, Mattace‐Raso, Sijbrands, & Zoccali, 2014; Glassock, Warnock, & Delanaye, 2017), resulting in elderly people being particularly susceptible to developing chronic kidney disease (CKD). The prevalence of CKD—defined as a glomerular filtration rate (GFR) inferior to 60 ml/min/1.73 m^2^—was thus more than 30% in a European cohort of patients over 70 years old (Ebert et al., 2016; Glassock et al., 2017). Nonspecific morphologic changes are associated with aging kidneys and designated by the generic term “nephrosclerosis.” Histologically, this is characterized by nephron loss, global glomerulosclerosis (GS > 10%), arteriosclerosis, tubular atrophy, and interstitial fibrosis (>5%) (Denic, Glassock, & Rule, 2016; O'Sullivan, Hughes, & Ferenbach, 2017).

Consequent to the rapid growth in the proportion of elderly people throughout the world (“European Commission: The 2015 ageing report. Eur Econ 3:424,” European Commission, 2015; GA, Atlanta, 2013), an increase in the number of CKD diagnoses would appear to be inevitable. However, more than a third of people exhibit no change in GFR with age (Bolignano et al., 2014; Lindeman, Tobin, & Shock, 1985), suggesting that age‐related renal impairment could be preventable. Gender, ethnicity, genetic factors, and comorbidities are known to influence renal outcomes (Choudhury & Levi, 2011; Smyth, Duffy, Maxwell, & McKnight, 2014), but other factors remain to be clarified.

The impact of advanced glycation end‐products (AGEs) on CKD, especially through binding to their main receptor RAGE (receptor for AGEs), has received significant research attention (Clarke, Dordevic, Tan, Ryan, & Coughlan, 2016; Stinghen, Massy, Vlassara, Striker, & Boullier, 2016). The AGEs form a heterogeneous group of molecules resulting from permanent binding of reducing sugars to a range of amino‐compounds. Their endogenous formation occurs under various conditions such as hyperglycemia and oxidative stress, but also aging. Their presence is moreover clearly identified in foods: Daily intake of *Nɛ*‐carboxymethyllysine (CML, the most studied AGE) can be as high as 252 µg/kg body weight in a typical European diet (Tessier & Birlouez‐Aragon, 2012). Evidence has recently accumulated incriminating the endogenous AGE/RAGE axis in age‐related diseases. RAGE is a multiligand, transmembrane receptor activating major pro‐inflammatory and pro‐oxidative signaling pathways. Its expression in numerous cell types increases with aging and pathological conditions such as diabetes, but a role for this receptor has been postulated in the premature dysfunction of several organs, even in the absence of diabetes (Frimat et al., 2017; Ramasamy, Shekhtman, & Schmidt, 2016). The impact of chronic exposure to dietary AGEs on aging remains poorly studied, however.

Considering the preferential accumulation of CML in the kidneys under a CML‐enriched diet (Li et al., 2015; Tessier et al., 2016) and studies linking dietary AGEs and kidney damage (Feng et al., 2007; Zheng et al., 2002), we hypothesized that kidneys are target organs for accelerated aging induced by AGE/RAGE interactions. In order to study this question, histologic markers of renal aging were analyzed in 2‐month‐old male wild‐type (WT) and RAGE^−/−^ C57Bl/6 mice fed a control or a CML‐enriched diet over 18 months.

## 2 RESULTS

### 2.1 Prolonged intake of a CML‐enriched diet results in renal accumulation of glycation products

**Figure 1:**
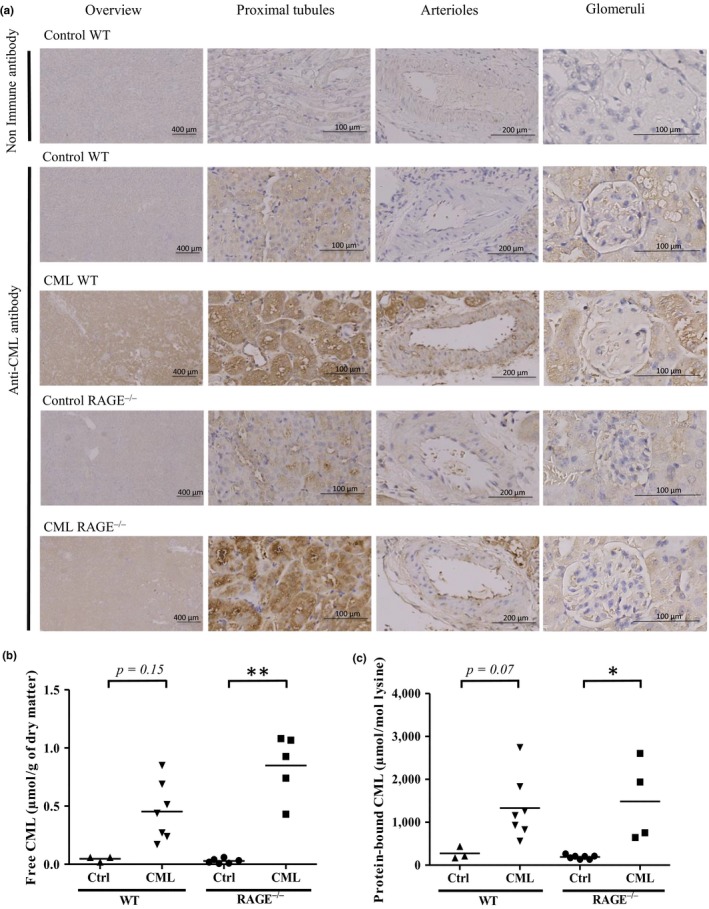
CML accumulation in the kidneys of WT and RAGE^−/−^mice was diet‐dependant. (a) Representative localization of protein‐bound CML studied by IHC on kidney sections showed that mice fed a CML‐enriched diet exhibited predominantly tubular staining. From left to right: low magnification, high magnification on proximal tubules, arterioles and glomeruli. (b‐c) Quantification by HPLC‐MS/MS of (b) free and (c) protein‐bound CML in kidneys. *p < 0.05, **p < 0.01, Kruskal–Wallis test

A diffuse antibody‐mediated CML staining was observed in the kidneys of all WT mice but was more intense in mice fed a CML‐enriched diet compared with control mice. CML staining predominated in the proximal tubular cells and along the internal wall of arteries. We found similar results in the kidneys of RAGE^−/−^ mice suggesting that CML accumulation from the CML‐enriched diet is governed by RAGE‐independent mechanisms (Figure 1a). These results were confirmed using liquid chromatography with tandem mass spectrometric (HPLC‐MS/MS) detection in kidney samples. The mean levels of free CML were higher among the mice fed the CML‐enriched diet, both in WT (0.05 ± 0.02 and 0.45 ± 0.2 µmol/g of dry matter for control and CML diet, respectively: *p* = 0.15) and RAGE^−/−^ mice (0.03 ± 0.02 and 0.85 ± 0.3 µmol/g of dry matter for control and CML diet, respectively: *p < *0.01). These differences between groups were also observed for concentrations of protein‐bound CML (275 ± 145 and 1,331 ± 740 µmol/mol lysine for control and CML WT mice, respectively: *p = *0.07; 190 ± 47 and 1,486 ± 947 µmol/mol lysine for control and CML RAGE^−/−^ mice, respectively: *p < *0.05), thereby suggesting a RAGE‐independent mechanism of CML formation (Figure 1b‐c).

### 2.2 Prolonged intake of a CML‐enriched diet does not significantly affect renal aging

**Figure 2:**
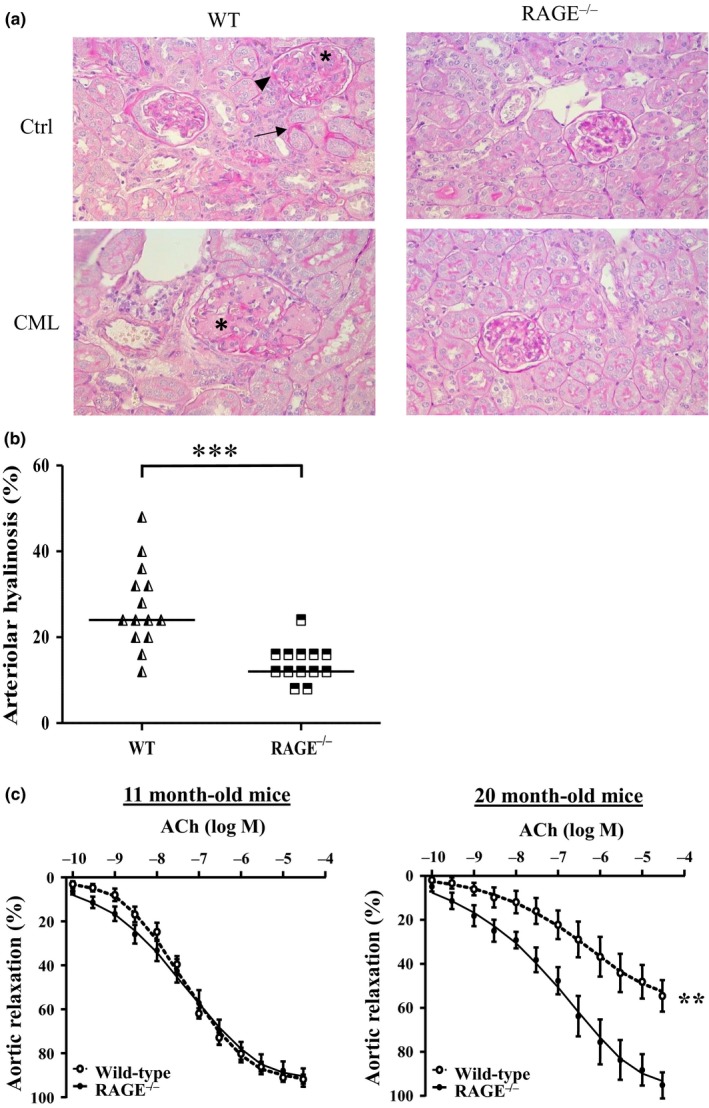
Vascular aging of renal arterioles was reduced in RAGE^−/−^ mice. (a) Representative PAS staining of paraffin‐embedded kidney sections of WT (*left panels*) and RAGE^−/−^ mice (*right panels*) fed a control (*upper panels*) or CML‐enriched diet (*lower panels*) revealed the presence of hyalinosis (arrowheads), tubules undergoing tubular atrophy (arrows) and glomerulosclerosis (asterisks) (x400). (b) Kidney arteriolar hyalinosis determined by PAS staining in WT and RAGE^−/−^ mice and (c) alteration in endothelium‐dependant relaxation in 11‐month‐old and 20‐month‐old WT and RAGE^−/−^ mice fed a control diet. Aortic relaxation is expressed as mean ±SEM (*n* = 5–6 mice in duplicate). **p < 0.01, ***p < 0.001, unpaired *t* test on area under the curve

Prior to assessing the effects of a CML‐enriched diet and RAGE invalidation on renal aging, we first ensured that weight, glycaemia, cholesterolemia, and triglyceridemia were comparable between mice fed the control and CML‐enriched diets. None of the WT or RAGE^−/−^ mice became diabetic, dyslipidemic, or obese under the influence of a CML‐enriched diet (Supporting Information Table S1). Renal parameters were then studied, and lesions of arteriolar hyalinosis, tubular atrophy, interstitial fibrosis, and GS (histological markers of nephrosclerosis, Figure 2a) were all assessed “blind.”.

Renal weight, plasma creatinine, and urea were not statistically different between control and CML groups, in either WT or RAGE^−/−^ mice (data not shown). We analyzed the expression of *Kim‐1* and *Ngal* as sensitive markers of kidney injury associated with CKD progression in humans (Alderson et al., 2016): The expression of these genes was also unaffected by the CML‐rich diet (Supporting Information Figure S1a‐b).

The assessment of nephrosclerosis markers at 20 months of age gave similar results: The CML‐rich diet had no significant effect on arteriolar hyalinosis, tubular atrophy, or fibrosis score (Supporting Information Figure S1c‐e). There was a minor worsening of GS under the influence of the CML‐rich diet in WT mice (Supporting Information Figure S1f‐h): Only 6.5% glomeruli lacked any sign of sclerosis in CML WT mice against 31%, 61% and 67% in control WT, control RAGE^−/−^ and CML RAGE^−/−^, respectively; the median GS score and percentage of global GS—the end‐stage of glomerular lesion—also followed this trend (CML WT>control WT>control and CML RAGE^−/−^). These differences were not significant, however, in contrast to the results observed when we compared WT with RAGE^−/−^ mice. In order to analyze the impact of the RAGE invalidation on renal aging, we thus focused on differences between WT and RAGE^−/−^ mice, irrespective of diet.

### 2.3 RAGE knockout mice are protected against nephrosclerosis lesions

All mice had hyaline deposits on the arteriole walls (10% to 48% of studied vessels), but the median percentage of vessels with hyaline deposits was higher in WT than in RAGE^−/−^ mice (24% vs. 12%, respectively, *p* < 0.001) (Figure 2b). In order to study the evolution of endothelial function with age, we measured the percentage of aortic relaxation mediated by different concentrations of acetylcholine (Ach) in middle‐aged (11‐month‐old) or old (20‐month‐old) WT and RAGE^−/−^ mice. No difference was observed between 11‐month‐old WT and RAGE^−/−^ mice (92 ± 7 and 91% ± 2% of max relaxation, respectively). At 20 months old, however, WT mice exhibited reduced responsiveness to Ach (55% ± 2% of max. relaxation vs. 11‐month‐old WT mice, *p* < 0.001) in contrast to RAGE^−/−^ mice with conserved endothelial function (95% ± 1% of max. relaxation) (Figure 2c).

**Figure 3:**
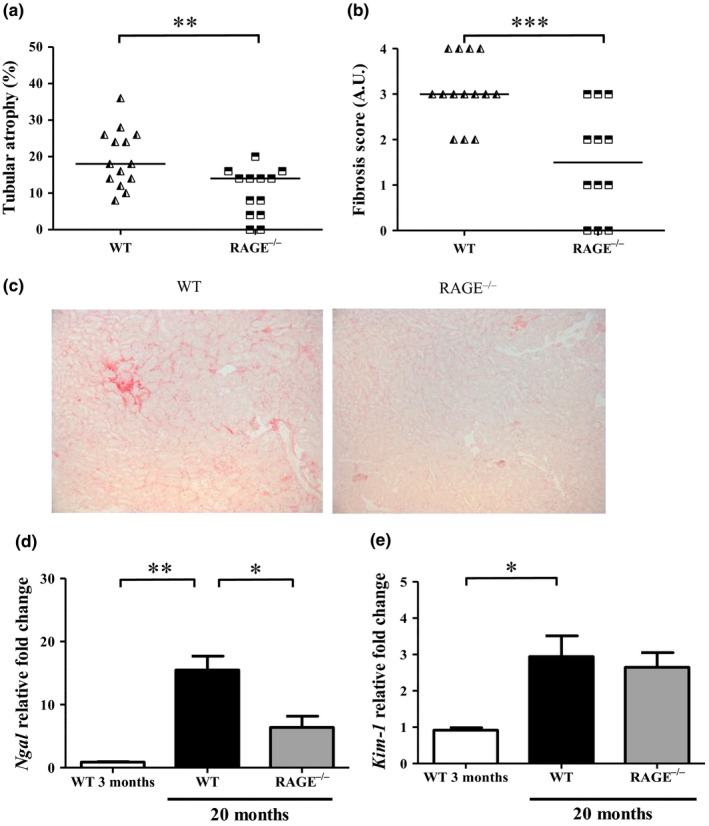
RAGE^−/−^ mice were protected against tubulo‐interstitial aging. Quantification of (a) tubular atrophy was determined by PAS staining and (b) interstitial fibrosis by Sirius red staining. (c) Representative Sirius red staining of control and CML WT and RAGE^−/−^ mice at low magnification (x 100). (d‐e) Expression of kidney injury markers *Ngal* (d) and *Kim‐1* (e) in renal tissue from 3‐ or 20‐month‐old WT and RAGE^−/−^ mice (mean ±SEM, *n* = 3 for 3‐month‐old mice and *n* = 10 for 20‐month‐old mice). *p < 0.05, **p < 0.01, ***p < 0.001, unpaired *t* test or Kruskal–Wallis test for multiple comparisons

Both tubular atrophy and interstitial fibrosis lesions were also more present in WT than in RAGE^−/−^ mice. The median percentage of tubular atrophy was 18% and 14% (*p* < 0.01), and the median fibrosis scores were 3 and 1 A.U. (*p* < 0.001) in WT and RAGE^−/−^ mice, respectively (Figure 3a‐b). Fibrosis was identified in renal interstitium of all WT mice, but was often mild to absent in RAGE^−/−^ mice (Figure 3c). The *Ngal* expression, more specifically associated with tubulointerstitial damage (Rysz, Gluba‐Brzózka, Franczyk, Jabłonowski, & Ciałkowska‐Rysz, 2017), was significantly higher in WT mice (∼15.5 ± 6.6) than RAGE^−/−^ mice (∼6.4 ± 5.6, *p < *0.05). Comparing old WT with old RAGE^−/−^ mice, there was no difference in the *Kim‐1*expression (Figure 3d‐e).

**Figure 4:**
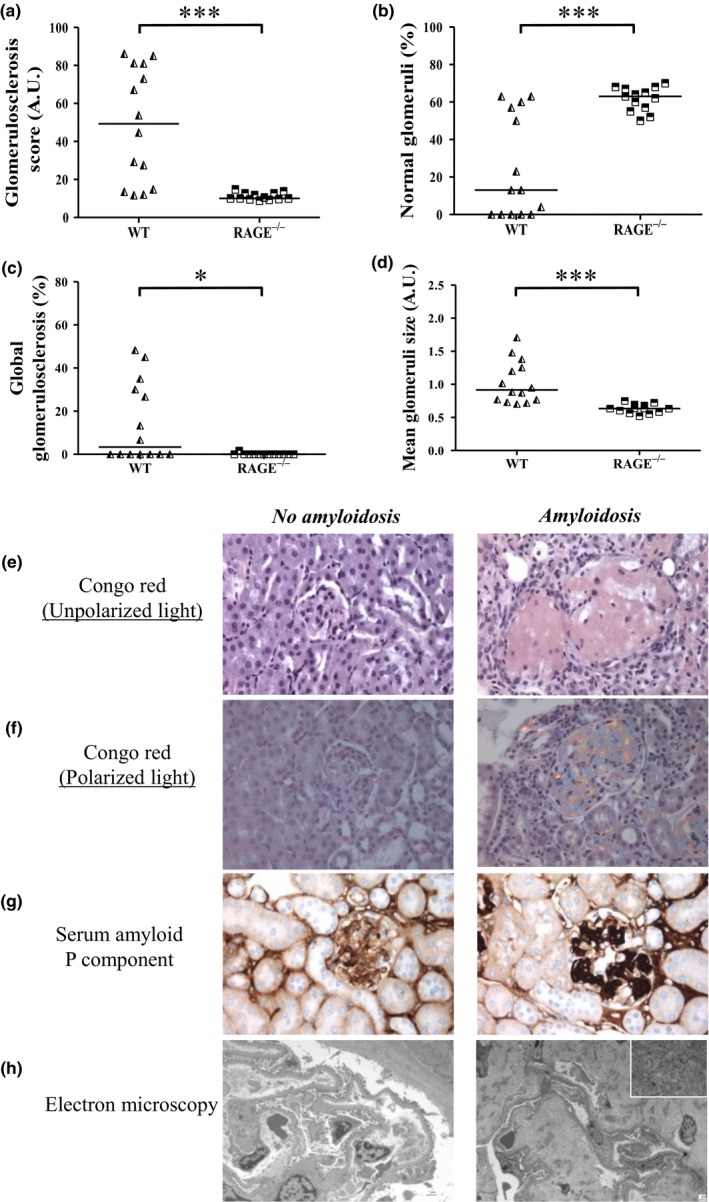
Amyloidosis‐linked glomerulosclerosis was largely prevented in RAGE^−/−^ mice. Quantification of GS in paraffin‐embedded kidney sections indicated (a) the GS score (60 glomeruli/mouse) which was used to determine (b) the percentage of normal glomeruli (glomeruli with GS score of 0) and of (c) global GS (glomeruli with a GS score of 100). (d) Mean glomeruli size (30 glomeruli/mouse). Determination of amyloidosis‐positive lesions in paraffin‐embedded kidney sections after (e) Congo red staining under nonpolarized light (x 400) and (f) polarized light showing birefringence (x400), (g) Immunohistochemistry of Serum Amyloid P component (SAP, x 400) and (h) electron microscopy showing the absence in control WT mouse (*left panel*, x 1,000) and the presence in CML WT mouse (*right panel*, x 1670 low magnification, x 21,560 high magnification in the frame) of amyloid fibrils. *p < 0.05, ***p <0.001, Mann–Whitney test, representative images

The median GS score at 20 months of age, calculated after histological evaluation of sclerosis intensity of 60 glomeruli per mouse, was higher in WT mice (49.3 A.U.) than in RAGE^−/−^ mice (10 A.U., *p* < 0.001). Only 13% of the glomeruli lacked any sign of sclerosis in WT mice against 63% in RAGE^−/−^ mice (*p* < 0.001). The mean percentage of global GS (glomeruli with a GS score of 100) was 14.6% in WT mice, while no global GS was observed in RAGE^−/−^ mice (Figure 4a‐c).

### 2.4 RAGE knockout prevents senile amyloidosis

The GS appeared nodular with developing hypertrophic glomeruli. The mean size of the glomeruli was significantly larger in WT than in RAGE^−/−^ mice (1.0 ± 0.3 and 0.6 ± 0.1 A.U., respectively: *p* < 0.001, Figure 4d). Birefringence under polarized light, obtained after Congo Red staining, suggested that this nodular sclerosis was at least partly caused by amyloidosis. This was confirmed by the presence of serum amyloid protein (SAP) in immunohistochemistry assays, and the identification of 5–10 nm fibrils by electron microscopy (Figure 4e‐h). Thus, 8 of the 14 (57%) WT mice were amyloidosis positive against only 1 of the 13 (8%) RAGE^−/−^ mice (7 of the 9 amyloid mice were fed the CML‐rich diet, Supporting Information Figure S2a).

**Figure 5:**
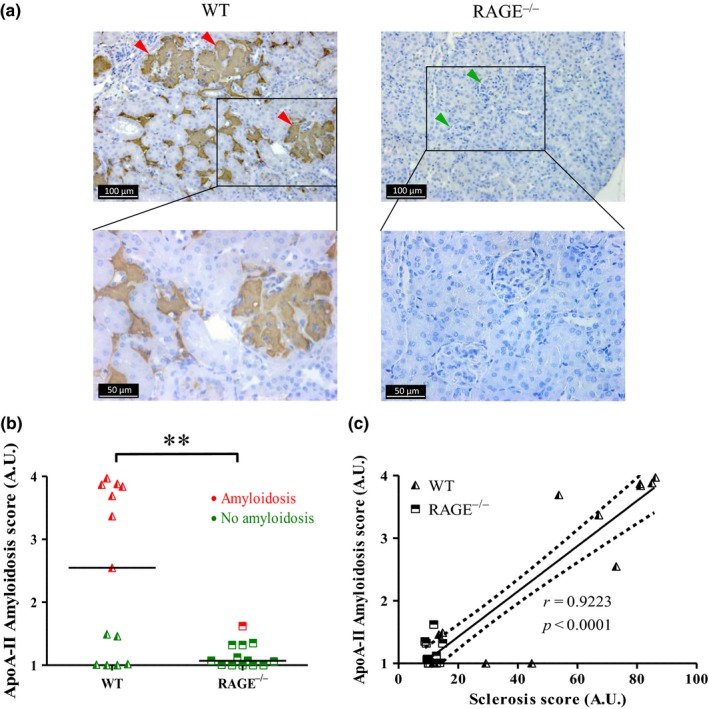
AApoA‐II was largely absent in RAGE^−/−^ mice. AApoA‐II in kidney tissue sections from 20‐month‐old WT mice (*left panel*) and RAGE^−/−^ mice (*right panel*) were immunostained using an anti‐ApoA‐II antibody (a) green arrowheads, no deposits; red arrowheads, extensive deposits (representative image). (b) Scoring of ApoA‐II amyloid deposits obtained from IHC and calculated from the grading of 100 glomeruli per mouse, where 1 = no deposit, 2 = small, 3 = moderate and 4 = extensive deposits. Red dots, amyloidosis‐positive mice; green dots, amyloidosis‐negative mice. (c) Linear regression analysis between AApoA‐II and sclerosis scores obtained for all four conditions, y = 0.036 x + 0.70, r = 0.9223, dotted line: 95% confidence intervals (C). **p < 0.01, unpaired *t* test

A positive ApoA‐II protein staining occurred in the same locations as the amyloid deposits, suggesting an ApoA‐II amyloidosis (AApoA‐II) thought to be related to aging processes in mice (Kitagawa et al., 2003). The ApoA‐II deposition was mainly localized in glomeruli, with various intensities according to the glomeruli and individual mice (Figure 5a). Interstitial AApoA‐II was also observed when the vast majority of glomeruli showed extensive deposits, suggesting a more advanced stage of amyloidosis. Other proteins known to be amyloidogenic were tested (serum amyloid A, involved in chronic inflammatory diseases, and peptides β1–40 and β1–42, responsible for amyloid plaques in Alzheimer's disease), but none were correlated with the identification of amyloidosis (data not shown). The WT mice had a median AApoA‐II score significantly higher than RAGE^−/−^ mice which had no or very few deposits (2.5 ± 1.3 vs. 1.1 ± 0.2 A.U., respectively: *p < *0.01; Figure 5b). A trend also suggested greater AApoA‐II deposition in CML WT than in control WT mice (1.2 A.U.), but this trend was not observed when RAGE^−/−^ groups were compared (Supporting Information Figure S2b). The GS intensity was correlated with AApoA‐II score (*r* = 0.92, *p < *0.0001, Figure 5c).

### 2.5 RAGE knockout mice exhibited less inflammation and were better protected against oxidative stress

**Figure 6:**
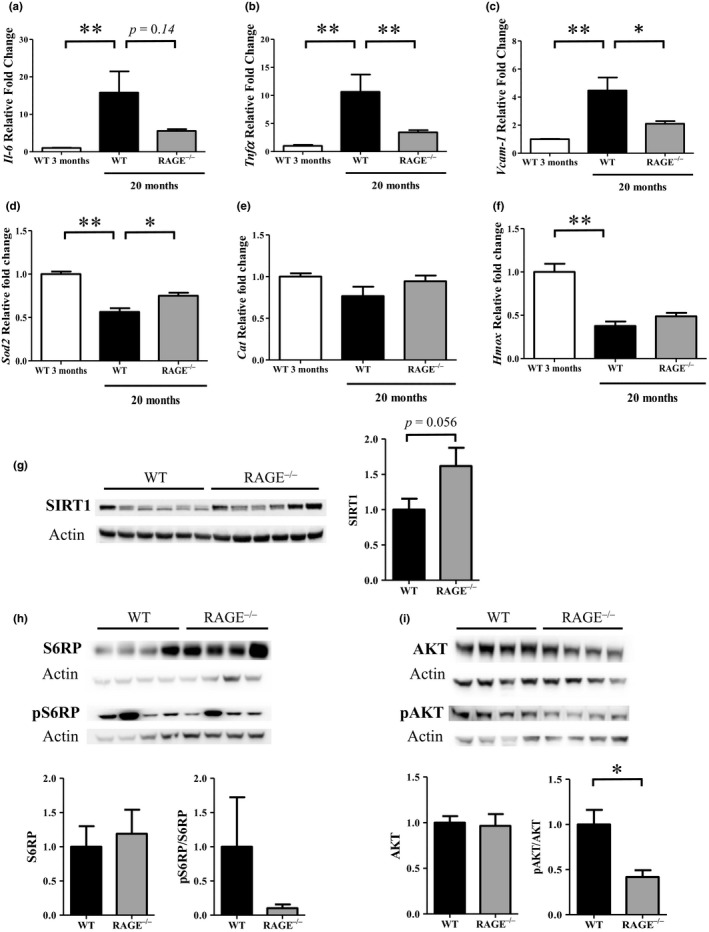
Inflammation and oxidation markers were decreased in RAGE^−/−^ mice. Inflammation markers (a) *Il‐6*, (b) *Tnfα* and (c) *Vcam‐1* and oxidation markers (d) *Sod2*, (e) *Cat* and (f) *Hmox*mRNA expression were measured in renal tissue from 3‐ or 20‐month‐old WT and RAGE^−/−^ mice (mean ±SEM, *n* = 3 for 3‐month‐old mice and *n* = 10 for 20‐month‐old mice). Representative western blot and quantification of protein levels of (g) SIRT1 (mean ±SEM, *n* = 10), (h) pS6RP (Ser235/236)/S6RP and (i) pAKT (Ser473)/AKT in kidney extracts of 22‐ to 26‐month‐old mice (mean ±SEM, *n* = 4). *p < 0.05, **p < 0.01, Mann–Whitney test or Kruskal–Wallis test for multiple comparisons

Compared to 3‐month‐old WT mice, the mRNA levels of *Il‐6*, *Tnf‐α* and *Vcam‐1* increased, respectively, ~15.8‐, 10.6‐ and 4.5‐fold in 20‐month WT mice and ~5.6‐, 3.4‐ and 2.1‐fold in RAGE^−/−^ mice (Figure 6a‐c). The increase in these markers was significant in old WT mice but not in old RAGE^−/−^ mice, suggesting a similar pro‐inflammatory profile in young WT and old RAGE^−/−^ mice. Expression of the antioxidant genes, *Sod2* (a mitochondrial manganese‐dependent superoxide dismutase), *Cat* (a peroxide hydrogen detoxifier) and *Hmox* (known to increase under influence of oxidative stress and recently described as ameliorating the oxidative stress‐induced endothelial senescence (Luo et al., 2018)), was also analyzed. While *Cat* expression did not change with age or genotype, *Sod2* expression was significantly lower in old WT mice when compared with young WT (0.56 ± 0.1, *p* < 0.01) and old RAGE^−/−^ mice (0.75 ± 0.1, *p* < 0.05). An important decrease in *Hmox* was also observed in both old WT (0.38 ± 0.2) and RAGE^−/−^ (0.49 ± 0.1) mice but was only significant in the former (*p < *0.01) (Figure 6d‐f).

As a first step to elucidate the mechanisms underlying this potential protective effect of RAGE knockout, and in light of our results on inflammation and oxidation markers, we focused on SIRT1 (associated with longevity and known to decrease with age (Yuan et al., 2016)) and the AKT/mTor pathway [involved in kidney disease (Kajiwara and Masuda, 2016)]. SIRT1 was higher in RAGE^−/−^ than WT mice (1.6‐fold ±0.8, *p* = 0.056, Figure 6g). Conversely, the S6 subunit of the 40S ribosome (pS6RP or S235/236), a mTORC1 activity indicator and, more significantly, pAkt (S473, a mTORC2 activity indicator) were lower in old RAGE^−/−^ than WT mice (0.1 ± 0.11 and 0.42 ± 0.15, respectively, *p* < 0.05) (Figure 6h‐i).

## 3 discussion

Although the small sample sizes necessitate caution in interpreting our results, we here report that RAGE knockout mice developed significantly less nephrosclerosis and senile amyloid lesions than WT mice, suggesting a key role for this receptor in the renal aging process. Prolonged exposure to a CML‐enriched diet was associated with CML accumulation in kidneys, but there was no evident relationship between these deposits and renal injuries; CML accumulation was RAGE‐independent and thus could not explain the differences between WT and RAGE^−/−^ mice in kidney function/structure. We found lower levels of inflammation and oxidative stress in RAGE^−/−^ mice, which may contribute to our proposed protective effect of RAGE invalidation through modulation of SIRT1 expression and the AKT/mTOR pathway.

In accordance with other studies, CML accumulated in kidneys of animals fed a CML‐enriched diet (Li et al., 2015; Tessier et al., 2016). We confirmed the RAGE‐independent nature of this accumulation (Tessier et al., 2016), and also separately quantified both free/dipeptide CML and protein‐bound CML. Since dietary CML cannot enter the circulation in protein‐bound form (Alamir et al., 2013), protein‐bound CML must result from endogenous formation. This could be the consequence of oxidative stress, a condition thought to favor CML production from dicarbonyl compounds and known to occur in aging and CKD (Roca, Grossin, Chassagne, Puisieux, & Boulanger, 2014; Stinghen et al., 2016). Thus, ingested CML could activate other, RAGE‐independent oxidative stress pathways, such as the epidermal growth factor receptor pathway (Chen et al., 2010), and/or participate in protein synthesis as described with noncanonical amino acids in artificial systems (Calve, Witten, Ocken, & Kinzer‐Ursem, 2016). Surprisingly, we did not observe a significant negative effect of a CML‐enriched diet on nephrosclerosis lesions in comparison with the significant positive impact of RAGE invalidation. Only GS and ApoAII amyloid deposits were higher (but not significantly so) in the CML WT versus control WT mice. While there is no published study establishing a direct link between AGE accumulation and renal aging, these results nevertheless seem to run counter to a body of related evidence. A potential deleterious effect of AGE accumulation can be inferred from diabetic nephropathy (Choudhury & Levi, 2011; Vlassara et al., 1994), in which CML deposits are, however, more pronounced. Moreover, an AGE‐rich diet has been associated with proteinuria and structural changes in the kidneys of rodent models, whereas an AGE‐poor diet induced fewer histological lesions and conserved renal function (Feng et al., 2007; Zheng et al., 2002). We hypothesize that any effect of dietary AGEs could be obscured by the relatively advanced age of our mice. Indeed, the majority of old control WT mice presented significant lesions of all renal structures and a third exhibited AApoA‐II, suggesting that aging is important in this regard.

Among the age‐related renal lesions that we reported, this observation of a link between nodular GS and AApoA‐II is interesting. While AApoA‐II is very uncommon in humans, it is described as a senile renal marker in mice and its prevalence varies according to genotype (Kitagawa et al., 2003). This age‐related susceptibility to AApoA‐II in mice may be explained by two characteristics of this protein. First, human ApoA‐II is dimerized while the murine ApoA‐II is monomeric and hence less stable. Second, ApoA‐II has a higher methionine content in mice than humans and methionine is an amino acid known to be prone to oxidation (Gursky, 2014). It has been shown that the lipid peroxidation of high‐density lipoproteins (HDLs, of which ApoA‐II is a major constituent) induced oxidation of methionine components (Anantharamaiah et al., 1988); consequently, the 3D structure of ApoA‐II could change and alter its affinity for HDLs. With subsequent dissociation from HDLs, ApoA‐II could unfold until its aggregation and subsequent nucleation, the first step in amyloid fibrils formation (Wong, Binger, Howlett, & Griffin, 2010). Promoting oxidation and lipid peroxidation, aging, and RAGE expression together could thus favor structural changes and the deposition of ApoA‐II in the kidneys, causing amyloidosis followed by GS. In a susceptible model such as C57BL6 mice, AApoA‐II appears to be a useful marker of aging that is highly reduced when RAGE is knocked‐out, and would further appear to be accelerated by prolonged intake of a CML‐enriched diet.

Not only AApoA‐II, but all the nephrosclerosis lesions assessed here were reduced in RAGE^−/−^ mice. The involvement of this receptor has already been reported in different kidney diseases, especially diabetic nephropathy. Indeed, genetic deletion or pharmacologic blockage of RAGE was associated with a decrease of renal lesions in different animal models of diabetes (Kaida et al., 2013; Myint et al., 2006; Vlassara et al., 1994). Independent of the underlying etiology, RAGE has been also implicated in CKD and its attendant cardiovascular complications through the formation of AGEs and the promotion of oxidative stress induced by renal failure itself (Stinghen et al., 2016). Our work here has illustrated that, even in the absence of prior kidney disease, RAGE could be involved in “normal” renal aging. The pro‐aging mechanisms induced by RAGE are multiple and especially include the perpetuation of a pro‐inflammatory phenotype and ROS generation; in accordance with this, RAGE^−/−^ mice here presented less inflammation and were better protected against oxidative stress. Previous studies have shown that a deficiency in SOD2 results in oxidative stress, interstitial inflammation, and accelerated renal senescence in a murine model (Rodriguez‐Iturbe et al., 2007). Thus, the smaller decrease of *Sod2* expression in RAGE^−/−^ mice with age could suggest enhanced protection against renal aging. Concurrently, and in accordance with previous studies showing that knockout or inhibition of RAGE prevented CML‐ or LPS‐induced reduction in SIRT1 expression (Grossin et al., 2015; Huang et al., 2015), SIRT1 expression was here downregulated in old WT compared with old RAGE^−/−^ mice. Interestingly, reduced renal inflammation and fibrosis have been reported in genetic or pharmacological boosting of SIRT1 models (Hong et al., 2018; Morigi, Perico, & Benigni, 2018); on the other hand, its deletion was associated with an aggravation of kidney injuries such as GS (Chuang et al., 2017). Together, these results suggest a potentially major role for SIRT1 in renal aging. Moreover, our observation of decreased levels of phosphorylated AKT and S6RP in RAGE^−/−^ mice could indicate a role for RAGE invalidation in a pro‐longevity phenotype and renoprotection. Indeed, mTOR inhibition is the only therapeutic intervention that leads to enhanced health and life span in all studied models (Johnson, Rabinovitch, & Kaeberlein, 2013; Weichhart, 2018), and downregulation or inhibition of mTORC1 prevents GS, glomerular hypertrophy macrophage infiltration, and other lesions in murine models of diabetic nephropathy (Kajiwara and Masuda, 2016). We thus suggest that the greater inflammation we observed in old WT mice compared with old RAGE^−/−^ mice could be the result of AKT‐ and/or SIRT1‐dependant NF‐κB pathway activation (Kane, Shapiro, Stokoe, & Weiss, 1999; Kauppinen, Suuronen, Ojala, Kaarniranta, & Salminen, 2013).

Taken together, our results point to a role for the RAGE receptor and its attendant signaling pathways in regulating inflammation, elevation of which is a recognized hallmark of aging (López‐Otín, Blasco, Partridge, Serrano, & Kroemer, 2013) and central to so‐called inflamm‐aging (Franceschi et al., 2000). They also suggest that RAGE^−/−^ mice present a pro‐longevity phenotype, as evidenced by their apparently attenuated renal aging. Further studies, in particular using tissue culture techniques, will be helpful not only in determining the mechanisms governing the pathways involved in this phenotype, but also in determining the potential utility of RAGE inhibitors in reducing renal aging in particular, and perhaps other age‐related phenomena.

## 4 EXPERIMENTAL PROCEDURES

### 4.1 Animal experimentation

All animals were housed in the experimental research department of Lille University (Agreement number: C59–35010). They were maintained at constant temperature and humidity in a room with a 12‐hr light cycle. All experiments were conducted in accordance with institutional guidelines and French Ministry of Agriculture recommendations for the care and use of laboratory animals, and with the approval of the Charles Darwin Ethics Committee for animal experimentation (number #00822.01). The male WT (Janvier Laboratories, Le Genest‐St‐Isle, France) and RAGE^−/−^ (from Pr. A.M. Schmidt, New York University) C57Bl/6 mice (6–10/group) received either a control diet incorporating unglycated bovine serum albumin (control‐BSA), or an isocaloric diet enriched with glycated BSA rich in CML (CML‐BSA). Mice were fed ad libitum for 18 months (2 months old at beginning of protocol). Three‐ or 11‐month‐old mice fed control‐BSA were used as controls to analyze baseline renal and endothelial function. Old 22‐ to 26‐months‐old WT and RAGE^−/−^ mice fed a standard diet were used for western blot analysis.

CML‐BSA was prepared as previously (Grossin et al., 2015). After glycation (16 hr, 37°C), 37%–40% of the lysine moieties were found to be modified, 80%–99% of which (on a molar basis) had been modified to CML. The control‐BSA was prepared under the same conditions, but glyoxylic acid was omitted. The control‐BSA and CML‐BSA preparations (diluted in water) were added to mouse powdered standard diet (A04, Safe, Augy, France) at 200 μg CML/g_food_. The concentration of protein, fatty acids, fiber, and carbohydrate did not differ between control‐BSA and CML‐BSA diets. A basal concentration of 17.5 ± 0.7 µg CML/g_food_ was determined in control‐BSA diet. Diets were renewed every 7 days, and remaining food was weighed to assess mean daily CML intake. Mice were weighed every 3 months. At 20 months, surviving mice (6 control WT, 8 CML WT, 8 control RAGE^−/−^ and 5 CML RAGE^−/−^) were culled and blood samples collected by inferior vena cava puncture. Kidneys were halved longitudinally and immediately frozen for biochemical and chemical analyses, or preserved in FAA (Formaldehyde, Alcohol, Acetic acid), RNA later and glutaraldehyde for electron microscopic analyses.

### 4.2 Immunohistochemistry

Four micrometer kidneys sections were deparaffinized and rehydrated by successive 3‐min washes in xylene then ethanol (100% and 95%, respectively). Standard procedures were followed up for antigen retrieval (Target Retrieval Solution, Dako) and blockage steps (kit Vectastain ABC Elite Peroxydase), except for ApoA‐II. Antigen retrieval was performed as previously (Kai et al., 2012): briefly, sections were first treated with proteinase K for 3 min. (Dako, ready‐to‐use) followed by an ethylenediaminetetraacetic acid (EDTA) bath (0.01 M, pH6 at 121°C for 10 min.) then a formic acid bath (70% for 5 min. at room temp.). Nonspecific fixation sites were blocked with rabbit or goat serum 5% (Sigma, R1933, G9023), while for ApoA‐II assays, endogenous peroxydases were blocked using a Dual Endogenous Enzyme Block (Dako, S200380) and endogenous biotins with an Endogenous Avidin/Biotin Blocking Kit (Abcam, ab64212).

Primary antibodies (18 hr, 4°C) were as follows: anti‐CML (rabbit Ig; 1:1000; Abcam ab27684), anti‐SAP (rabbit Ig, Biocare PP132AA), and anti‐ApoA‐II (goat, 2 µg/ml, Santa Cruz sc‐23609). Corresponding nonimmune Igs served as negative controls. After incubation (1 hr, room temp.) with Streptavidin HRP Conjugate (ImmunoReagents, Ba‐103‐HRPX), sections were stained using DAB Substrate (Cell Signaling, Kit #8059P) and counterstained using hematoxylin solution (Sigma Aldrich, GHS316) before mounting under coverslips.

### 4.3 HPLC‐MS/MS

A high‐pressure liquid chromatography (HPLC)‐triple quadrupole mass spectrometer (MS/MS) was used to quantify CML and lysine by isotope dilution in kidney extracts (Vantage LC‐MS/MS, ThermoFisher Scientific). Frozen kidneys were lyophilized (Alpha 1–2 LD plus, Bioblock scientific) for 24 hr at −40°C and −0.1 mbar. Twenty mg of kidney was suspended in 10% trichloroacetic acid (Sigma Aldrich) for 30 min. on ice. After centrifugation at 21 000 G for 10 min. at 4°C, the supernatant (free CML) and the pellet (protein‐bound CML) were separately incubated in NaBH_4_ (83 mM) for 2 hr at room temp. The reaction was stopped by adding HCl 37% (1:1). The samples were then incubated at 110°C for 21 hr and a 200µl aliquot evaporated using a SpeedVac Concentrator (Thermo Scientific). Isotope‐labeled internal standards were added to the final volume of 200µl of aqueous nonafluoropentanoic acid (NFPA, 5 mM, Sodipro), as follows: CML‐d_2_ 0.05 mM (Polypeptide Group) or Lysine‐^15^N_2_ 50 mM (Cortecnet). The samples were then filtered through 0.45 µm nylon filters (Sodipro). Separation was performed on a Hypercarb column (100 × 2.1 mm, 5 µm particle size; ThermoFisher Scientific) equipped with a guard column (10 × 2.1 mm, 5 µm particle size). Injection volume was 10µl, column temp. 10°C, and compounds were eluted with a binary mobile phase at 0.2 ml/min: Mobile phase A was a 10 mM aqueous solution of the ion‐pairing agent NFPA, while mobile phase B was LC‐MS grade acetonitrile (Sigma Aldrich). The elution gradient was as follows: 0–9 min 100%–75% A; 9–11 min 40% A; 11–13 min 40%–100% A; 13–21 min 100% A. The heated electrospray interface was operated in positive ionization mode (3,500 V, 250°C, sheath and auxiliary gas flows at 50 and 20 arbitrary units, respectively). The MS/MS transitions monitored were as follows: *m/z* 205‐130 and *m/z* 207‐130 for CML and CML‐d_2_, and *m/z* 147‐130 and *m/z* 149‐131 for lysine and Lysine‐^15^N_2_, respectively. Quantification was performed using the ratio of responses for the unlabeled molecules and their respective isotope‐labeled internal standards in a 9‐point calibration covering the concentration ranges 0–0.2 mM (CML) and 0–20 mM (Lysine).

### 4.4 Histological analyses

Two sections were stained (3 or 5 µm according to purpose, at an interval of 200 µm) from the halves of paraffin‐embedded kidneys retained for histological analyses: by Periodic Acid Schiff (PAS) to assess GS, tubular atrophy and arteriolar hyalinosis; by Sirius red to evaluate interstitial fibrosis under polarized light; and by Congo red to detect amyloid deposits under optical and polarized light. Samples were labeled without reference to treatment group, and all parameters (defined below) were analyzed “blind” by a qualified anatomopathologist and nephrologist using an optical microscope (Leica DM5500 B).

#### 4.4.1 Definitions

*Arteriolar hyalinosis* appeared as a pink hyaline material in arteriolar walls after PAS staining. For each mouse, 25 arterioles (10–15/section) were analyzed and described as positive or negative.

*Tubular atrophy* was defined by an attrition of the brush border of the tubular epithelium, a reduced tubular size and a thickening of the tubular basement membrane. For each mouse, 50 tubules (25/section) were analyzed and described as positive or negative.

*Interstitial fibrosis* was defined by a red‐signal in Sirius red staining in the cortex (excepting the physiological staining in the border of the vessels). For each mouse, this parameter was graded thus: grade 0, no fibrosis; grade 1, at least 1 focal fibrosis lesion; grade 2, at least 2 focal or fibrosis lesions. The scores attributed by the two examiners were summed.

*Glomerulosclerosis (GS)*, identified as an increased mesangial matrix, was assessed on 60 superficial and juxtamedullary glomeruli (30/section) for each mouse. This parameter was scored between 0% and 100% (intervals of 10%) for each glomerulus. A GS score was expressed as the mean for each mouse. Normal glomeruli yielded a GS score of 0 while global GS was defined by the maximum possible score of 100, indicating glomeruli were completely sclerotic.

To assess the size of glomeruli, 6 fields/section were studied for each mouse. The area of each glomerulus was measured using ImageJ.

*Amyloidosis* was defined by positive Congo red staining. Amyloid deposits in glomeruli were graded thus: grade 1, no deposit; grade 2, small amount; grade 3, moderate amount; grade 4, extensive deposits. An amyloidosis score was adapted from the literature and represented the mean score of a section for 100 glomeruli analyzed (Luo et al., 2015).

### 4.5 Endothelial dysfunction: aortic reactivity

Aortic ring relaxation was investigated in 11‐ and 20‐month‐old WT and RAGE^−/−^ mice fed the control diet. Aortic ring responsiveness was tested as previously (Grossin et al., 2015) in an automated dual small‐vessel myograph (ADInstruments, Chalgrove, UK). Briefly, aortas were contracted by adding 3 × 10^‐6^ M phenylephrine (Sigma) to standard Krebs solution in an organ bath (37°C, 5% CO_2_ in O_2_). Once the contraction phase had stabilized, the endothelium‐dependent relaxation (EDR) was tested by the cumulative addition of acetylcholine (ACh, 10^−10^ × 10^−4^ M; Sigma). Relaxation responses were expressed as a percentage of the maximal phenylephrine‐induced contraction.

### 4.6 RT‐qPCR

RNA extraction from half‐kidneys was performed with a commercial kit (Qiagen). ARN was quantified by spectrophotometry at 260 nm (Biotech Spec‐nano, Shimadzu Biotech) and quality analyzed by RNA Standard Sens kit (BIO‐RAD). For RT‐qPCR, amplification of cDNA was performed with the following probe mixes (ThermoFisher): *Kim‐1*(Mm00506686), *Ngal* (Mm01324470), *Il‐6*(Mm00446190_m1), *Tnfα*(Mm00443258_m1)*, Vcam‐1* (Mm01320970_m1), *Sod2* (Mm01313000_m1), *Cat* (Mm00437992_m1), and *Hmox*(Mm00516005_m1). The mean cycle threshold (CT) values for both the target and internal control (*Ppia,*Mm02342430_g1) were determined for each sample. The fold change in the target gene, normalized to *Ppia* and relative to the expression of WT mice fed the control diet, was calculated as 2^−ΔΔCT^.

### 4.7 Western blot

Kidney tissues were homogenized in RIPA buffer in the presence of protease and phosphatase inhibitors in 1.5% β‐mercaptoethanol using a precellys homogenizer. After centrifugation (19,000 g for 10 min at 4°C), supernatants were collected for protein analysis. Protein concentration was determined by the Bradford method (Bio‐Rad protein assay dye, Bio‐Rad): 20 µg of protein was separated using bolt 4%–12% Bis‐Tris plus (Invitrogen) gels, or NuPAGE 7% Tris‐Acetate gels (ThermoFisher) for larger molecular weights and was transferred to a polyvinylidene difluoride membrane (Bio‐Rad). Nonspecific binding sites were blocked for 1 hr at 20°C with 5% milk or BSA, according to the antibody, in Tris‐buffered Saline with 0.05% Tween 20 (TBS‐T). Membranes were incubated with anti‐SIRT1 (ab7343, Abcam, 1:900), anti‐FoxO3A (12829, Cell Signaling, 1:1000), anti‐pFoxO3A (Ser253, Ab47287, Abcam, 1:750), anti‐S6RP (2317, Cell Signaling, 1:1000), anti‐pS6RP (Ser235/236, 4858, Cell Signaling, 1:2000), anti‐Akt (4691, Cell Signaling, 1:1000), anti‐pAkt (Ser473, 9271, Cell Signaling 1:1000), or anti‐Actin (A2066, Sigma Aldrich, 1:2000) overnight at 4°C followed by washing and incubation with horseradish peroxidase‐ (HRP‐) conjugated anti‐rabbit (7074, Cell Signaling, 1:5000) or anti‐mouse (7076, Cell Signaling, 1:2000). After washing, chemiluminescent reaction was induced using Clarity Western ECL Substrate (Bio‐Rad) and detection performed using a Fusion FX Spectra imager (Vilber). Bands were normalized to Actin levels using ImageJ.

### 4.8 Biologic parameters

Cholesterol, triglycerides, urea, and fasting glucose serum levels were measured with a biochemistry analyzer VetTest® (IDEXX laboratory) according to manufacturer's instructions. Serum creatinine levels were measured by an enzymatic colorimetric assay with an autoanalyzer (COBAS 8000 modular analyzer series; kits #CREP2–05168589190).

### 4.9 Statistical analysis

Statistical analysis was performed using GraphPad Prism 5.0 (La Jolla, CA, USA). A Kruskal–Wallis test followed by a Dunn's multiple comparison test was applied for multiple comparisons; otherwise, a two‐tailed, unpaired *t* test was performed for samples with n∼10 and a Mann–Whitney test was performed for smaller samples. A *p*‐value < 0.05 was considered as significant in either case (*α* = 0.05).

## CONFLICT OF INTEREST

The authors declare no conflict of interest.

## AUTHORS’ CONTRIBUTION

Study design: MF and EB. Provided RAGE‐KO mice: AMS. Performed research: TT, VQ, MD, MH, FD, CL and TB. Histological data analyses: VQ, VG and MF. Statistical analyses: TT and FG. MH provided English proofreading assistance. All authors discussed the data and approved the submission.
