# Supplementary material for: Knockout of receptor for advanced glycation end‐products attenuates age‐related renal lesions
Source: Aging Cell. 2019 Feb 22;18(2):e12850. doi: 10.1111/acel.12850 (PMC6413655; doi:10.1111/acel.12850)
Supplement: Supplementary file 1 [file ACEL-18-e12850-s001.pdf]

Supplementary Table 1:

	WT		RAGE$^{-/-}$	
	Ctrl	CML	Ctrl	CML
Fasting blood glucose (g/L)	1.11 ± 0.30	1.02 ± 0.11	1.07 ± 0.14	1.03 ± 0.10
Serum cholesterol (mg/L)	0.55 ± 0.16	0.74 ± 0.19	0.81 ± 0.22	0.63 ± 0.22
Triglyceridemia (mg/L)	0.93 ± 0.32	0.72 ± 0.31	0.83 ± 0.31	0.58 ± 0.14
Mice weight (g)	33.70 ± 1.87	33.74 ± 2.47	33.15 ± 1.28	33.32 ± 2.05
Kidneys weight (mg)	223 ± 32	227 ± 27	204 ± 21	217 ± 37

Supplementary Table 1. Control and CML WT and RAGE$^{-/-}$ mice have similar metabolic conditions. Values of serum glycaemia (g/L), cholesterolemia (mg/L), triglyceridemia (mg/L) and mice weight (g) at last follow-up showed no signs of obesity or diabetes. Kidney weights (g) measured before longitudinal cut (n=4-5). Kruskal-Wallis test.

Fig. S1:

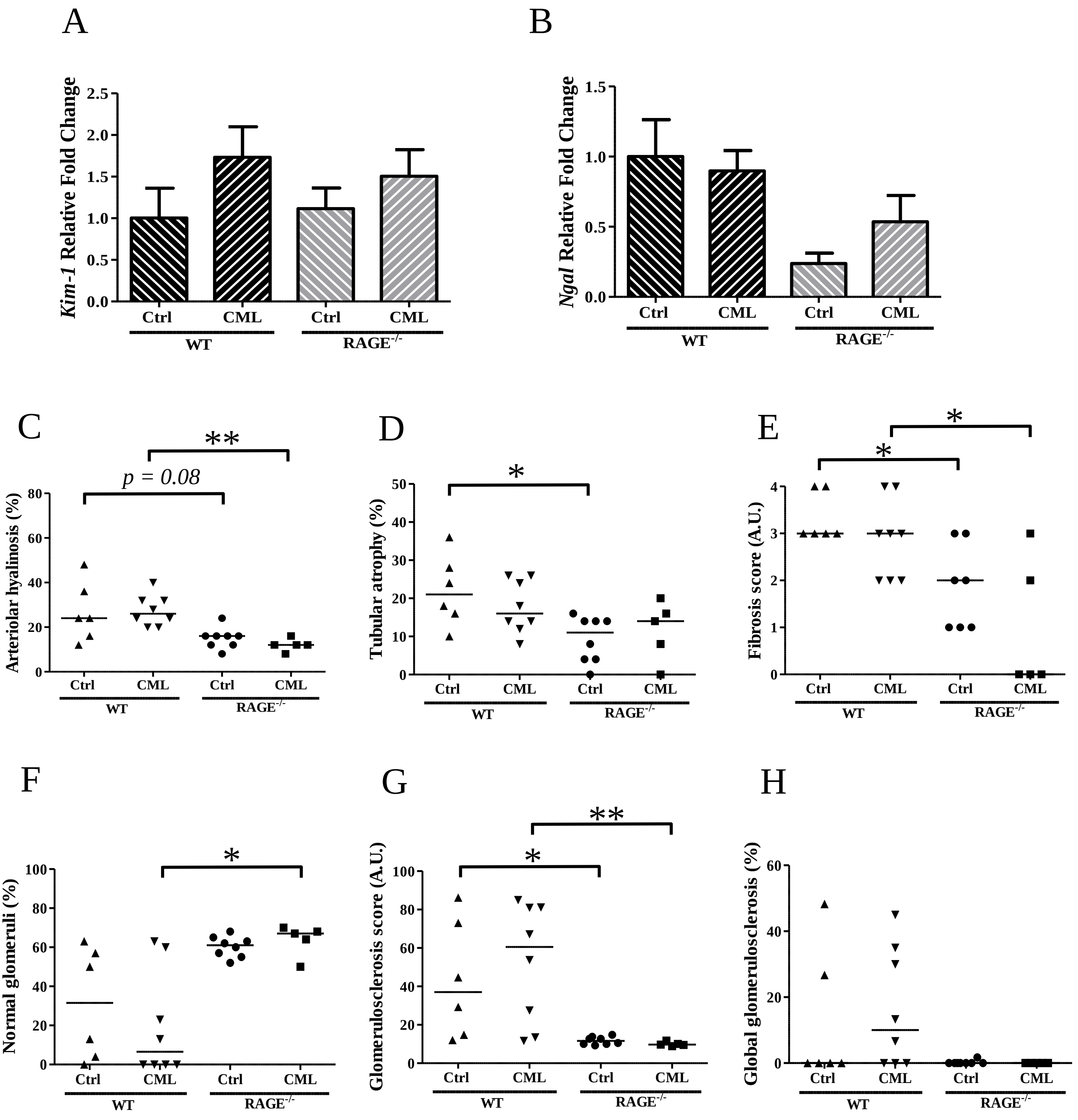

Supplementary figure 1. CML has a limited impact on renal suffering markers and histological lesions. (**A-B**) Expression of kidney injury markers *Kim-1* (**A**) and *Ngal* (**B**) in renal tissue from 20 month-old control and CML WT and RAGE$^{-/-}$ mice (mean ± SEM, n=5). Histological lesions in paraffin-embedded kidney sections such as (**C**) arteriolar hyalinosis and (**D**) tubular atrophy were determined by PAS staining and (**E**) fibrosis by Sirius red staining. Quantification of glomerulosclerosis (60 glomeruli/mouse) gave (**F**) the normal glomeruli percentage (glomeruli with GS score of 0), (**G**) the GS score and (**H**) the global GS percentage (glomeruli with a GS score of 100). *p <0.05, **p <0.01, Kruskal-Wallis test.

Fig. S2:

A

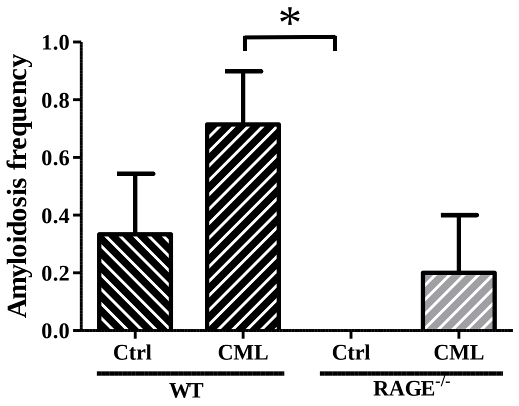

B

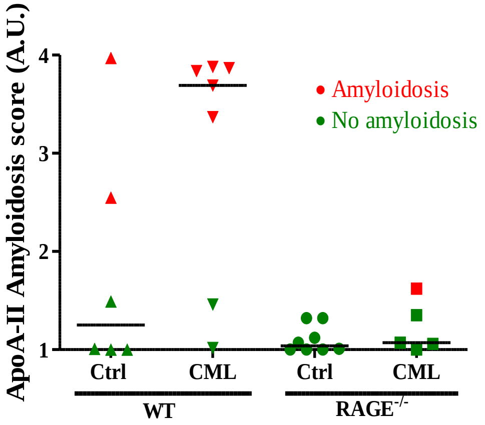

Supplementary figure 2. RAGE$^{-/-}$ mice are protected against ApoA-II amyloidosis. (**A**) Frequency of renal amyloidosis in each group. (**B**) Scoring of ApoA-II amyloid deposits obtained from IHC in control and CML WT and in control and CML RAGE$^{-/-}$ mice (n=5-8). *p <0.05, Kruskal-Wallis test.
